# Optimal cut-points of different anthropometric indices and their joint effect in prediction of type 2 diabetes: results of a cohort study

**DOI:** 10.1186/s12889-018-5611-6

**Published:** 2018-06-05

**Authors:** Neda Zafari, Mojtaba Lotfaliany, Mohammad Ali Mansournia, Davood Khalili, Fereidoun Azizi, Farzad Hadaegh

**Affiliations:** 1 Prevention of Metabolic Disorders Research Center, Research Institute for Endocrine Sciences, Shahid Beheshti University of Medical Sciences, Tehran, Iran; 2 Non-Communicable Disease Control, School of Population and Global Health, University of Melbourne, Melbourne, VIC Australia; 3 Department of Epidemiology and Biostatistics, School of Public Health, Tehran University of Medical Sciences, Tehran, Iran; 4 Endocrine Research Center, Research Institute for Endocrine Sciences, Shahid Beheshti University of Medical Sciences, Tehran, Iran

**Keywords:** BMI, Waist circumference, Waist to height ratio, Obesity, Cohort study, Joint effect

## Abstract

**Background:**

To determine the anthropometric indices that would predict type 2 diabetes (T2D) and delineate their optimal cut-points.

**Methods:**

In a cohort study, 7017 Iranian adults, aged 20–60 years, free of T2D at baseline were investigated. Using Cox proportional hazard models, hazard ratios (HRs) for incident T2D per 1 SD change in body mass index (BMI), waist circumference (WC), waist to height ratio (WHtR), waist to hip ratio (WHR), and hip circumference (HC) were calculated. The area under the receiver operating characteristics (ROC) curves (AUC) was calculated to compare the discriminative power of anthropometric variables for incident T2D. Cut-points of each index were estimated by the maximum value of Youden’s index and fixing the sensitivity at 75%. Using the derived cut-points, joint effects of BMI and other obesity indices on T2D hazard were assessed.

**Results:**

During a median follow-up of 12 years, 354 men, and 490 women developed T2D. In both sexes, 1 SD increase in anthropometric variables showed significant association with incident T2D, except for HC in multivariate adjusted model in men. In both sexes, WHtR had the highest discriminatory power while HC had the lowest. The derived cut-points for BMI, WC, WHtR, WHR, and HC were 25.56 kg/m^2^, 89 cm, 0.52, 0.91, and 96 cm in men and 27.12 kg/m^2^, 87 cm, 0.56, 0.83, and 103 cm in women, respectively. Assessing joint effects of BMI and each of the obesity measures in the prediction of incident T2D showed that among both sexes, combined high values of obesity indices increase the specificity for the price of reduced sensitivity and positive predictive value.

**Conclusions:**

Our derived cut-points differ between both sexes and are different from other ethnicities.

**Electronic supplementary material:**

The online version of this article (10.1186/s12889-018-5611-6) contains supplementary material, which is available to authorized users.

## Background

Diabetes is the most prevalent metabolic disorder in the world [1]. It is a coronary heart disease equivalent [2–4] and had a substantial global burden with 680 disability-adjusted life years per 100,000 people in 2010 [1]. By 2035 the prevalence of type 2 diabetes (T2D) will increase by 55% worldwide with an alarming pace in developing countries such as those in the Middle East and North Africa region (approximately 96.2% increase in prevalence in 20 years) [5].

One of the major modifiable risk factors of T2D is obesity [6–10]. Despite clear evidence linking obesity to various health outcomes including cardiovascular diseases (CVD), obesity has been a puzzling condition for clinicians because it is quite heterogeneous [11] and the existing evidence regarding the suitable anthropometric index to be used as a screening test in each sex is controversial. On the one hand, the literature supports the application of central obesity, such as waist to height ratio (WHtR) over general obesity indicators in assessing T2D risk [12, 13]. On the other hand, there are studies that use body mass index (BMI) as their main obesity index in predicting T2D [14–16]. Several studies have been conducted to obtain the optimum cut-points of anthropometric indices [17–22]. However, these studies were mainly conducted in Asians, European and American Caucasians. Understandably, the results are not necessarily generalizable to other ethnicities [23]. Therefore, WHO has emphasized the need for prospective studies to derive and validate ethnic-specific cut-points of body fat composition indices to predict CVD and T2D [24].

Located in the Middle East, Iran suffers from high incidence and prevalence of T2D [10, 25]. Yet, data regarding appropriate anthropometric cut-points in the Iranian population using prospective studies is limited [26].

In the current study, we decided to determine the sex-specific independent and combined risk of different anthropometric indices in the prediction of T2D in the cohort of Tehran Lipid and Glucose Study (TLGS) with over 12 years of follow-up; furthermore, we compared the discriminatory power of adiposity measures and delineated their optimal cut-points in this population.

## Methods

### Study design and sample

The TLGS, an ongoing prospective population-based study being performed on a representative sample of the Tehran population, aims to determine the prevalence and incidence of non-communicable diseases and their risk factors. Detailed descriptions of the TLGS have been reported elsewhere [27]. In brief, one baseline (1999–2001) and 4 follow-up examinations at triennial intervals have been carried out until January 2015. Those who had cancer, end-stage renal disease or cirrhosis were excluded from the TLGS at the baseline examination [28]. For the current study, 10,727 participants, aged 20–60 years [8569 people from the baseline examination (1999–2001) and 2158 new participants recruited from the second phase (2001–2005)], were selected. Subjects with prevalent T2D at their baseline examination [*N* = 806, excluding those with known T2D using anti-diabetic medications (*N* = 276), 32 participants had isolated high fasting plasma glucose (FPG), 95 subjects had high FPG levels while their 2 h-post-challenge plasma glucose (2 h-PCPG) was missing, 202 participants had isolated high 2 h-PCPG and 201 had combined high FPG and high 2 h-PCPG], no data on baseline variables (*N* = 1342) or not any follow-up data (*N* = 1562) were excluded; leaving 7017 participants (2988 men, 4029 women) to include in the analyses as respondents (response rate = 7017/(10,727–806) × 100 = 70.7%). Furthermore, to compare the discriminatory power of anthropometric indices and to delineate their optimal cut-points for the prediction of T2D in each sex, the analyses were performed only among those who participated in the last follow-up phase and those with incident T2D during the follow-ups (*N* = 5738, 2419 men, 3319 women) (Additional file 1).

### Clinical and laboratory measurements

Using a pretested questionnaire, a trained interviewer collected information regarding demographic data, drug history and family history of T2D. Weight was measured, with subjects minimally clothed without shoes, using digital scales (Seca 707: range 0–150 kg) and recorded to the nearest 1 kg. Using a tape meter, height was measured in a standing position without shoes, while shoulders were in normal alignment; waist circumference (WC) was measured at the umbilical level and that of the hip (HC) at the widest girth of the hip over light clothing, without any pressure to the body surface. Measurements were recorded to the nearest 1 cm. Body mass index (BMI) was calculated as weight (Kg) divided by height squared (m^2^). Waist to hip (WHR) and waist to height ratios (WHtR) were calculated as WC (cm) divided by HC (cm) and height (cm), respectively. Wrist circumference was measured with the anterior wrist surface facing up. The superior border of the tape meter was placed just distal to the prominences of the radial and ulnar bones, without any tape pressure over it; values were recorded to the nearest 0.1 cm.

After a 15-min rest in the sitting position, two measurements of systolic and diastolic blood pressure (SBP and DBP) were taken on the right arm, using a standardized mercury sphygmomanometer (calibrated by the Iranian Institute of Standards and Industrial Researches); the mean of the two measurements was considered as the participant’s blood pressure.

A blood sample was taken between 7:00 and 9:00 AM from all study participants, after 12 to 14 h of overnight fasting. All blood analyses were carried out at the TLGS research laboratory on the day of the blood collection. For all non-pharmacologically treated diabetic participants aged ≥20 years, an oral glucose tolerance test with 82.5 g of glucose monohydrate solution [equivalent to 75 g of anhydrous glucose; Cerestar EP, Spain] was performed; a second blood sample was obtained 2 h after glucose ingestion. Fasting plasma glucose (FPG) and 2 h-post-challenge plasma glucose (2 h-PCPG) were measured using an enzymatic colorimetric method with glucose oxidase; inter- and intra-assay coefficients of variation at baseline and follow-up phases were both less than 2.3%.

High-density lipoprotein cholesterol (HDL-C) was measured after precipitation of the apolipoprotein B containing lipoproteins with phosphotungstic acid. Triglycerides (TG) were assayed using glycerol phosphate oxidase. Both inter- and intra-assay coefficients of variation were less than 3 and 2.1% for HDL-C and TG, respectively. Analyses were performed using Pars Azmon kits (Pars Azmon Inc., Tehran, Iran) and a Selectra 2 auto-analyzer (Vital Scientific, Spankeren, Netherlands). Triglyceride to high-density lipoprotein cholesterol ratio (TG/HDL-C) was calculated by dividing TG to HDL-C. All samples were analyzed when internal quality control met the acceptance criteria.

### Definition of terms

Subjects who reported a parent or sibling with diabetes were considered as having a positive family history of T2D. Education level was classified in 3 categories: i) those who had studied less than 6 years, ii) those who had studied for 6–12 years, and iii) those with more than 12 years of education. In accordance with the definition provided by the American Diabetes Association, [29] participants were considered to have T2D if they met at least one of these criteria: FPG ≥7.0 mmol/L, or 2 h-PCPG ≥11.1 mmol/L or taking anti-diabetic medication. In addition, participants with missing data on 2 h-PCPG at follow-up who simultaneously had FPG levels< 5.05 mmol/L were considered free of T2D [30].

### Statistical methods

All statistical analyses were stratified by sex. Continuous variables were described as mean (standard deviation (SD)) or median (interquartile range (IQR)), and categorical variables were summarized as frequency (percentage). Baseline characteristics of men and women, as well as respondent and non-respondent groups, were compared using independent T-test, Mann-Whitney U test, and Chi-square test whichever indicated. The event date for diabetes cases was described as the middle-time between the date of follow-up visit at which diabetes was detected for the first time, and the most recent follow-up visit preceding the diagnosis; the follow-up time was considered as the difference between the calculated mid-time date and the date at which the subjects entered the study. For the censored subjects, the survival time was the interval between their first and last observation dates.

Cox proportional hazard models with age as the time scale [31] were used to estimate the hazard ratios (HRs) with 95% confidence intervals (95% CIs) for incident T2D per 1 SD increase in anthropometric indices in univariate (without adjustment for any other variables) and adjusted multivariable models. In model 1, the hazard ratio of interest was adjusted for education, family history of T2D, SBP, FPG, and TG/HDL-C. In model 2, the hazard ratio of interest was adjusted for variables included in model 1 plus wrist circumference as it has been shown to be a significant predictor of T2D in the TLGS adult population [32].

The proportional hazards assumption in the Cox models was assessed both graphically and using the Schoenfeld residual test. All proportionality assumptions were met. Collinearity was checked by estimating the first order correlation coefficients between variables used in each model as well as using correlation matrix of coefficients in the multivariable adjusted cox model [33]. There was no pair of variables with correlation coefficient of 0.80 or more in models. Receiver operating characteristics (ROC) curves were plotted to compare the discriminative power of different anthropometric indices for the prediction of incident T2D. The equality of the area under the ROC curves (AUCs) of different anthropometric indices was tested using the Stata command ‘roccomp’ [34]. Using the “R optimal cut-point package”, [35] cut-points for each variable were estimated by i) the maximum value of Youden’s index i.e., sensitivity+specificity-1, [36] ii) setting the sensitivity at 75%.

To examine the joint effects of BMI-WC, BMI-WHtR, BMI-WHR, and BMI-HC on the hazard of T2D in each sex, combined variables were created. To compare 4 categories, considering those with normal BMI-WC, BMI-WHtR, BMI-WHR, and BMI-HC as reference groups, using our cut-points derived from the fixed sensitivity at 75%, Cox proportional hazard models with age as the time scale were used in univariate and adjusted multivariable models. Model 1 was adjusted for education, family history of T2D, SBP, FPG, and TG/HDL-C. Model 2 was adjusted for variables included in model 1 and wrist circumference. All analyses were done using Stata (version 12.0) and R (version 3.4.3).

## Results

The study population consisted of 2988 men and 4029 women with the mean (SD) ages of 37.8 (10.2) and 37.3 (10.4) years, respectively. Baseline characteristics of respondents and non-respondents (eligible participants whose baseline or follow-up data were missing) are shown in Additional file 2. The only significant difference between these two groups was that respondents were 4 years older than their non-respondent counterparts. All other variables were generally the same in both groups. Table 1 depicts baseline characteristics of respondent men and women. Men were older and had higher levels of education, wrist circumference, SBP, FPG and log TG/HDL-C. Regarding anthropometric indices, men had higher WC and WHR, whereas, BMI, HC, and WHtR were higher in women. Frequency of baseline consumption of angiotensin-converting enzyme inhibitors (ACEIs), diuretics, corticosteroids and lipid-lowering drugs in men were 0.6, 0.5, 1.5, and 1.4%, respectively; the corresponding values for women were 0.7, 1.9, 1.9, and 2%, respectively.

**Table 1:** Baseline characteristics^a^ of respondents, Tehran Lipid and Glucose Study (1999–2015)

	Men (*N* = 2988)	Women (*N* = 4029)	Difference (CI)
Age (years)	37.8 (10.2)	37.3 (10.4)	0.5 (0.0;0.1)
Family History of T2D; No. (%)	790 (26.4)	1115 (27.7)	−1.2% (−3.3;0.8)
Education Level; No. (%)			
< 6 years	471 (15.8)	1127 (28.0)	−12.2% (− 14.1; − 10.3)
6–12 years	1888 (63.2)	2402 (59.6)	3.6% (1.2;5.9)
> 12 years	629 (21.1)	500 (12.4)	8.6% (7.8; 10.4)
Wrist Circumference (cm)	17.6 (1.0)	15.9 (1.0)	1.7 (1.6; 1.7)
WC (cm)	88.4 (11.1)	86.0 (12.3)	2.4 (1.8;2.9)
Height (cm)	171.1 (6.6)	157.4 (5.8)	13.7 (13.4;14.0)
WHtR	0.51 (0.06)	0.54 (0.08)	−0.03 (−0.033; −0.026)
BMI (kg/m^2^)	25.7 (4.1)	27.2 (4.9)	−1.5 (−1.7; −1.3)
HC(cm)	96.8 (7.2)	103.9 (9.5)	−7.1 (−7.6; −6.7)
WHR	0.91 (0.06)	0.82 (0.07)	0.08 (0.08;0.09)
SBP (mmHg)	116.0(14.1)	113.5 (15.6)	2.5 (1.8;3.3)
DBP (mmHg)	76.6 (10.3)	75.8 (10.3)	0.8 (0.3;1.3)
FPG (mmol/L)	5.00 (0.50)	4.91 (0.51)	0.09 (0.07;1.2)
Log TG/HDL-C	0.60 (0.71)	0.26 (0.69)	0.37 (0.34;0.40)

^a^For continuous variables, values are presented as mean (SD) and difference (95% CI) was estimated using linear regression models. Categorical variables are presented as frequency (percentage) and difference (95% CI) was estimated by logistic regression

*CI* confidence interval, *T2D* type 2 diabetes mellitus, *WC* waist circumference, *WHtR* waist to height ratio, *BMI* body mass index, *HC* hip circumference, *WHR* waist to hip ratio, *SBP* systolic blood pressure, *DBP* diastolic blood pressure, *FPG* fasting plasma glucose, *TG/HDL-C* triglyceride to high density lipoprotein cholesterol ratio

During a median follow-up (IQR) of 11.9 (4.6) years, 354 new cases of T2D in men and 490 ones in women were detected resulting in an annual crude incidence rate (95% CI) of 10.9 (9.8–12.1) and 11.1 (10.1–12.1) diabetes per 1000 person-years of follow-up in men and women, respectively. Table 2 illustrates sex-specific adjusted HRs (95% CIs) for incident T2D per 1 SD increase in anthropometric indices using the univariate and multivariable-adjusted Cox proportional hazard models. In men, in line with the univariate model, model 1 indicated statistically significant associations between all anthropometric measures and T2D incidence with HRs ranging from 1.25 for HC to 1.37 for BMI. Considering the wrist circumference as a surrogate of body frame in model 2, the hazardous association of all the obesity measures for development of T2D was shown except for HC (HR 0.92, 95% CI: 0.80–1.06). In women, similar to the univariate model, model 1 indicated statistically significant hazardous associations between all anthropometrics and incident T2D, with HRs ranging from 1.24 for HC to 1.51 for WHtR. However, after adjusting for wrist circumference in model 2, all the HRs decreased but still remained statistically significant.

**Table 2:** Associations between anthropometric indices and incident type 2 diabetes, Tehran Lipid and Glucose Study (1999–2015)

	Univariate model		Model 1^b^	Model 2^c^		
	HR^a^ (95% CI)	*P*-value	HR (95% CI)	*P*-value	HR (95% CI)	*P*-value
Men						
BMI (kg/m^2^)	1.69 (1.54–1.85)	< 0.001	1.37 (1.24–1.51)	< 0.001	1.48 (1.31–1.68)	< 0.001
WC (cm)	1.69 (1.52–1.87)	< 0.001	1.35 (1.21–1.50)	< 0.001	1.43 (1.26–1.64)	< 0.001
WHtR	1.66 (1.51–1.83)	< 0.001	1.36 (1.23–1.50)	< 0.001	1.39 (1.24–1.55)	< 0.001
WHR	1.52 (1.38–1.67)	< 0.001	1.27 (1.14–1.41)	< 0.001	1.26 (1.13–1.40)	< 0.001
HC (cm)	1.52 (1.38–1.67)	< 0.001	1.25(1.14–1.37)	< 0.001	0.92(0.80–1.06)	0.304
Women						
BMI (kg/m^2^)	1.74 (1.60–1.89)	< 0.001	1.42 (1.30–1.56)	< 0.001	1.41 (1.27–1.57)	< 0.001
WC (cm)	1.90 (1.74–2.08)	< 0.001	1.48 (1.34–1.64)	< 0.001	1.46 (1.30–1.64)	< 0.001
WHtR	1.93 (1.76–2.12)	< 0.001	1.51 (1.37–1.68)	< 0.001	1.47 (1.32–1.64)	< 0.001
WHR	1.57 (1.44–1.71)	< 0.001	1.30 (1.19–1.42)	< 0.001	1.27 (1.16–1.39)	< 0.001
HC (cm)	1.46 (1.35–1.58)	< 0.001	1.24(1.14–1.35)	< 0.001	1.11(1.00–1.24)	0.043

^a^Per 1 SD increase for each index (SD of WC = 11.13, BMI = 4.08, HC = 7.16, WHR = 0.06, WHtR = 0.06 in men and WC = 12.31, BMI = 4.90, HC = 9.51, WHR = 0.07, WHtR = 0.08 in women)

^b^Adjusted for age (in time-scale manner), education level, family history of type 2 diabetes, systolic blood pressure, fasting plasma glucose, and triglyceride to high-density lipoprotein cholesterol ratio

^c^Adjusted for model 1 variables and wrist circumference

*HR* hazard ratio, *CI* confidence interval, *BMI* body mass index, *WC* waist circumference, *WHtR* waist to height ratio, *WHR* waist to hip ratio, *HC* hip circumference

**Fig. 1:**
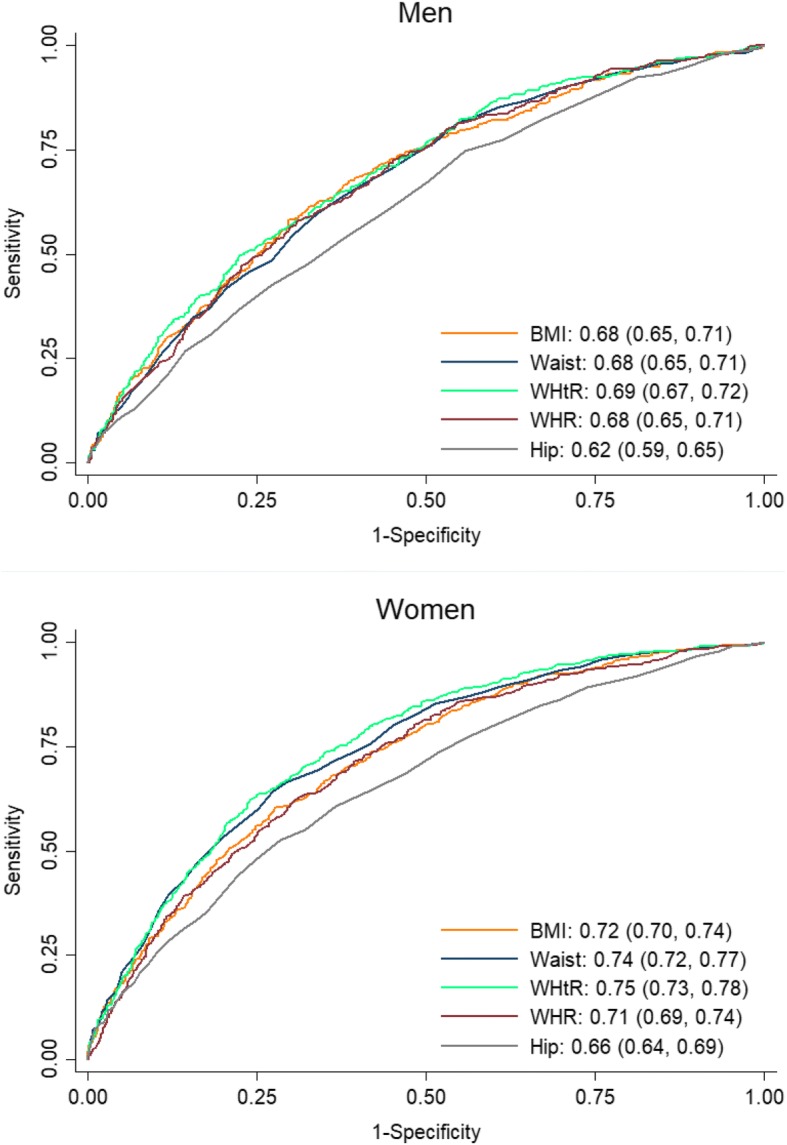
Receiver operating characteristic (ROC) curves, area under the curves (AUCs) and 95% confidence intervals for different anthropometrics by gender. *P*-values for AUCs comparison in men: BMI-WC: 0.51; BMI-WHR: 0.84; BMI-WHtR: 0.11; BMI-HC: < 0.001; WC-WHR: 0.80; WC-WHtR: 0.002; WC-HC: < 0.001; WHtR-HC: < 0.001; WHR-WHtR: 0.10; WHR-HC: < 0.001. *P*-values for AUCs comparison in women: BMI-WC: 0.002; BMI-WHR: 0.68; BMI-WHtR: < 0.001; BMI-HC: < 0.001; WC-WHR: 0.001; WC-WHtR: 0.004; WC-HC: < 0.001; WHtR-HC: < 0.001; WHR-WHtR: < 0.001; WHR-HC: 0.003. WHtR, waist to height ratio; WC, waist circumference; WHR, waist to hip ratio; BMI, body mass index

Fig. 1 shows sex-specific ROC curves and the AUCs (95% CIs) for different anthropometric indices. In both sexes, WHtR had the highest AUC (0.69, 95% CI: 0.67–0.72, in men and 0.75, 95% CI: 0.73–0.78, in women), whereas HC had the lowest values (0.62, 95% CI: 0.59–0.65, in men and 0.66, 95% CI: 0.64–0.69, in women).

Table 3 shows the sex-specific cut-points of different anthropometric indices for the prediction of incident T2D. In men, fixing sensitivity at 75%, the calculated cut-points for BMI, WC, WHtR, WHR, and HC were 25.56 kg/m^2^, 89 cm, 0.52, 0.91, and 96 cm while the corresponding values for the Youden’s index were 26.49 kg/m^2^, 87 cm, 0.54, 0.92, and 96 cm, respectively. In women, fixing sensitivity at 75%, the calculated cut-points for BMI, WC, WHtR, WHR, and HC were 27.12 kg/m^2^, 87 cm, 0.56, 0.83, and 103 cm while the corresponding values for the Youden’s index were 29.27 kg/m^2^, 91 cm, 0.56, 0.83, and 106 cm, respectively.

**Table 3:** Sex-specific diagnostic test performance of the anthropometric indices for incident type 2 diabetes, TLGS study

	Cut-point	No. of subjects above cut-point	No. of Incident T2D	Sensitivity (%)	Specificity (%)	PPV (%)
Men (*N* = 2419)						
BMI (kg/m^2^)						
Youden’s index	26.49	1043	243	67.7	61.4	23.3
Sensitivity = 75%	25.56	1258	269	74.9	52.0	21.4
WC (cm)						
Youden’s index	87.00	1415	292	81.3	45.5	20.6
Sensitivity = 75%	89.00	1254	265	73.8	52.0	21.1
WHtR						
Youden’s index	0.54	940	224	62.7	65.0	23.8
Sensitivity = 75%	0.52	1229	262	74.9	51.4	21.3
WHR						
Youden’s index	0.92	1122	246	71.9	55.5	21.9
Sensitivity = 75%	0.91	1247	267	75.2	50.3	21.4
HC (cm)						
Youden’s index	96.00	1418	268	74.6	44.2	18.9
Sensitivity = 75%	96.00	1418	268	74.6	44.2	18.9
Women (N = 3319)						
BMI (kg/m^2^)						
Youden’s index	29.27	1080	300	60.4	72.4	27.8
Sensitivity = 75%	27.12	1619	373	75.0	55.8	23.0
WC (cm)						
Youden’s index	91.00	1166	331	66.6	70.4	28.4
Sensitivity = 75%	87.00	1557	376	75.6	58.2	24.1
WHtR						
Youden’s index	0.56	1365	366	73.6	64.8	26.8
Sensitivity = 75%	0.56	1414	373	75.0	63.1	26.4
WHR						
Youden’s index	0.83	1502	358	73.0	59.1	23.8
Sensitivity = 75%	0.83	1589	371	75.0	56.7	23.3
HC (cm)						
Youden’s index	106.00	1338	302	60.8	63.3	22.6
Sensitivity = 75%	103.00	1833	367	73.8	48.0	20.0

*T2D* type 2 diabetes mellitus, *PPV* positive predictive value, *BMI* body mass index, *WC* waist circumference, *WHtR* waist to height ratio, *WHR* waist to hip ratio, *HC* hip circumference

Table 4, illustrates the joint effects of BMI and other obesity measures on the hazard of developing T2D, based on the cut-points derived from the fixed sensitivity at 75%, in men. As shown in the table, high BMI, whether alone or combined with high other obesity measures, had a significant risk for incident T2D both in univariate and multivariate models (except for high BMI-low HC). Among men population with normal BMI, only the presence of high WHR showed significant risk for incident T2D in the fully adjusted analysis (HR 1.55, 95% CI: 1.01–2.38).

**Table 4:** Joint-effects of BMI and other central obesity measures on the hazard of type 2 diabetes in men, TLGS study

	Incident T2D /total participants	Univariate model		Model 1^a^		Model 2^b^	
	(%)	HR (95% CI)	*P* Value	HR (95% CI)	*P*-value	HR (95% CI)	*P*-value
BMI (kg/m^2^)-WC(cm)							
< 25.56- < 89	70/1302 (5.38)	Reference	–	Reference	–	Reference	–
≥ 25.56- < 89	24/191 (12.57)	2.23(1.40–3.56)	0.001	1.75(1.10–2.80)	0.018	1.83(1.14–2.95)	0.012
< 25.56- ≥ 89	19/184 (10.33)	1.54(0.93–2.57)	0.093	1.04(0.62–1.74)	0.879	1.07(0.64–1.81)	0.778
≥ 25.56- ≥ 89	241/1311 (18.38)	3.13(2.39–4.09)	< 0.001	2.21(1.67–2.91)	< 0.001	2.38(1.75–3.25)	< 0.001
BMI (kg/m^2^)- WHtR							
< 25.56- < 0.52	73/1311 (5.57)	Reference	–	Reference	–	Reference	–
≥ 25.56- < 0.52	24/202 (11.88)	2.09(1.32–3.32)	0.002	1.73(1.08–2.75)	0.020	1.81(1.13–2.92)	0.014
< 25.56- ≥ 0.52	16/175 (9.14)	1.18(0.68–2.04)	0.549	0.80(0.46–1.40)	0.444	0.80(0.46–1.39)	0.437
≥ 25.56- ≥ 0.52	241/1300 (18.54)	2.98(2.28–3.88)	< 0.001	2.08(1.58–2.74)	< 0.001	2.20(1.64–2.97)	< 0.001
BMI (kg/m^2^)- WHR							
< 25.56- < 0.91	48/1116 (4.3)	Reference	–	Reference	–	Reference	–
≥ 25.56- < 0.91	44/354 (12.43)	2.89(1.92–4.36)	< 0.001	2.08(1.37–3.15)	0.001	2.20(1.43–3.38)	< 0.001
< 25.56- ≥ 0.91	41/370 (11.08)	2.05(1.34–3.13)	0.001	1.54(1.00–2.36)	0.047	1.55(1.01–2.38)	0.044
≥ 25.56- ≥ 0.91	221/1148 (11.85)	3.87(2.82–5.31)	< 0.001	2.70(1.95–3.74)	< 0.001	2.88(2.03–4.08)	< 0.001
BMI (kg/m2)- HC							
< 25.56- < 96	68/1147 (5.93)	Reference	–	Reference	–	Reference	–
≥ 25.56- < 96	23/158 (14.56)	1.98(1.23–3.18)	0.005	1.35(0.83–2.19)	0.224	1.41(0.86–2.30)	0.164
< 25.56- ≥ 96	21/339 (6.19)	1.03(0.63–1.69)	0.881	0.86(0.52–1.41)	0.564	0.92(0.55–1.51)	0.774
≥ 25.56- ≥ 96	242/1344 (18.01)	2.92(2.23–3.83)	< 0.001	2.15(1.63–2.84)	< 0.001	2.40(1.75–3.30)	< 0.001

^a^Adjusted for age (in time-scale manner), education level, family history of type 2 diabetes, systolic blood pressure, fasting plasma glucose, and triglyceride to high-density lipoprotein cholesterol ratio

^b^Adjusted for model 1 variables and wrist circumference

*HR* hazard ratio, *CI* confidence interval, *BMI* body mass index, *WC* waist circumference, *WHtR* waist to height ratio, *WHR* waist to hip ratio, *HC* hip circumference

Table 5, illustrates the joint effects of BMI and other obesity measures on the hazard of incident T2D, based on the derived cut-points from the fixed sensitivity at 75%, in women. Analyses revealed that high obesity measures whether alone or in combination with each other were generally associated with higher risk of developing T2D compared to the reference group. However, there were two exceptions: i) in those with high BMI-normal WHtR whose increased risk was not statistically significant in the multivariate models and ii) in those with normal BMI-high HC whose HRs were not significantly increased neither in univariate nor in multivariate models.

**Table 5:** Joint-effects of BMI and other central obesity measures on the hazard of type 2 diabetes in women, TLGS study

	Incident T2D /total participants	Univariate model		Model 1^a^		Model 2^b^	
	(%)	HR (95% CI)	*P* value	HR (95% CI)	*P*-value	HR (95% CI)	*P*-value
BMI (kg/m^2^)-WC(cm)							
< 27.12- < 87	77/1776(4.34)	Reference	–	Reference	–	Reference	–
≥ 27.12- < 87	44/389(11.31)	2.29(1.58–3.33)	< 0.001	1.89(1.29–2.75)	0.001	1.79(1.22–2.61)	0.003
< 27.12- ≥ 87	46/309(14.86)	2.66(1.83–3.87)	< 0.001	2.05(1.41–2.97)	< 0.001	1.99(1.36–2.89)	< 0.001
≥ 27.12- ≥ 87	323/1555(20.77)	3.76(2.89–4.89)	< 0.001	2.30(1.75–3.02)	< 0.001	2.06(1.53–2.77)	< 0.001
BMI (kg/m^2^)- WHtR							
< 27.12- < 0.56	81/1848(4.38)	Reference	–	Reference	–	Reference	–
≥ 27.12- < 0.56	42/463(9.07)	1.86(1.27–2.70)	0.001	1.45(0.99–2.11)	0.053	1.36(0.92–1.99)	0.113
< 27.12- ≥ 0.56	42/237(17.72)	3.13(2.13–4.60)	< 0.001	2.33(1.59–3.43)	< 0.001	2.32(1.58–3.42)	< 0.001
≥ 27.12- ≥ 0.56	325/1481(21.94)	4.02(3.11–5.21)	< 0.001	2.51(1.92–3.29)	< 0.001	2.27(1.70–3.03)	< 0.001
BMI (kg/m^2^)- WHR							
< 27.12- < 0.83	46/1464(3.14)	Reference	–	Reference	–	Reference	–
≥ 27.12- < 0.83	85/703(12.09)	3.25(2.25–4.67)	< 0.001	2.31(1.59–3.34)	< 0.001	2.11(1.44–3.08)	< 0.001
< 27.12- ≥ 0.83	77/621(12.40)	3.32(2.29–4.81)	< 0.001	2.55(1.75–3.71)	< 0.001	2.54(1.75–3.70)	< 0.001
≥ 27.12- ≥ 0.83	282/1241(22.72)	5.67(4.10–7.86)	< 0.001	3.33(2.37–4.67)	< 0.001	2.99(2.10–4.25)	< 0.001
BMI (kg/m2)- HC							
< 27.12- < 103	92/1627 (5.65)	Reference	–	Reference	–	Reference	–
≥ 27.12- < 103	37/214 (17.29)	2.39(1.63–3.52)	< 0.001	1.68(1.13–2.48)	0.009	1.56(1.05–2.31)	0.026
< 27.12- ≥ 103	31/458 (6.77)	1.09(0.72–1.64)	0.669	1.07(0.71–1.61)	0.721	1.02(0.67–1.53)	0.923
≥ 27.12- ≥ 103	330/1730 (19.08)	2.66(2.10–3.38)	< 0.001	1.79(1.40–2.30)	< 0.001	1.58(1.20–2.07)	0.001

^a^Adjusted for age (in time-scale manner), education level, family history of type 2 diabetes, systolic blood pressure, fasting plasma glucose, and triglyceride to high-density lipoprotein cholesterol ratio

^b^Adjusted for model 1 variables and wrist circumference

*HR* hazard ratio, *CI* confidence interval, *BMI* body mass index, *WC* waist circumference, *WHtR* waist to height ratio, *WHR* waist to hip ratio, *HC* hip circumference

Table 6 depicts the sensitivity, specificity and positive predictive value (PPV) of combinations of high BMI- high WC, high BMI- high WHtR, high BMI- high WHR, and high BMI- high HC using the derived cut-points by fixing sensitivity at 75% in men and women. As shown in the table, all the sensitivities fell to values under 70%, whereas the reported specificities were increased compared to the values displayed in Table 3. The overall effect of combining high measures together was a mild reduction in the PPVs.

**Table 6:** Sex-specific Prediction Accuracy of the Combined High Anthropometric Indices for Incident Type 2 Diabetes, TLGS study

	Sensitivity (%)	Specificity (%)	PPV (%)
Men (N = 2419)			
BMI ≥ 25.56- WC ≥ 89	68.1%	59.4%	18.4%
BMI ≥ 25.56- WHtR ≥ 0.52	68.1%	59.8%	18.5%
BMI ≥ 25.56- WHR ≥ 0.91	62.4%	64.8%	19.3%
BMI ≥ 25.56- HC ≥ 96	68.4%	58.2%	18.0%
Women (N = 3319)			
BMI ≥ 27.12- WC ≥ 87	65.9%	65.2%	20.8%
BMI ≥ 27.12- WHtR ≥ 0.56	66.3%	67.3%	21.9%
BMI ≥ 27.12- WHR ≥ 0.83	57.6%	72.9%	22.7%
BMI ≥ 27.12- HC ≥ 103	67.3%	60.4%	19.1%

*BMI* body mass index, *WC* waist circumference, *WHtR* waist to height ratio, *WHR* waist to hip ratio, *HC* hip circumference, *PPV* positive predictive value

## Discussion

Investigated in a large Iranian cohort study with 12 years of follow-up, all anthropometric variables showed significant association with incident T2D after adjustment for a wide set of covariates including a surrogate of body frame (wrist circumference) in both sexes, excluding hip circumference in men. Assessing their discriminatory power, in both sexes, WHtR performed the best, whereas HC showed the lowest prediction power. The derived cut-points of anthropometric indices for predicting the development of T2D in our population were generally different for men and women. Investigating the accuracy of combination of BMI and other obesity measures in predicting development of T2D revealed that in both sexes, the combined high values of general and central obesity indices increase the specificity for the price of reduced sensitivity and PPV.

In literature, there is almost unanimous agreement on the association of all general and central obesity measures with incident T2D in both sexes [15, 17, 19, 20, 22, 37–39]. However, controversy remains as to which index can predict incident T2D, independent of other obesity variables. While several studies suggest central obesity measures as the main obesity indices predicting T2D in men, [38–40]. there are investigations highlighting the role of general obesity as the optimal index [14, 15]. In line with other studies, we found that in both men and women, all anthropometric indices are almost similarly associated with T2D. Moreover, in our study, while wrist circumference acted as a positive risk factor for developing T2D in women, in men it showed a negative association. Similarly, Jahangiri et al. showed the positive association between wrist circumference and incident T2D in women [32].

With regards to HC, similar to other studies, [41–43] we showed the hazardous association of larger HC with development of T2D in both sexes in the multivariate analysis adjusted for traditional T2D risk factors; the association which reached to null after considering body frame (wrist circumference) in men. Moreover, further adjustment of HC for BMI, as suggested by many investigators, [44, 45] reversed this association only in women (HR 0.77, 95% CI: 0.65–0.92 in women, HR 0.82 95% C: 0.66–1.02 in men). Obesity is linked to higher risk of T2D [46]. However, there are studies which has reported a better metabolic profile in individuals who had more gluteofemoral mass for a given amount of abdominal fat [47–49]. The possible explanation for such effect is that HC is a presentation of several components namely the bone, the gluteal muscles, and the gluteal subcutaneous fat [50]. Each component plays its own role in the process of developing T2D. Gluteal muscles are one of the main sites of insulin receptors. Therefore, higher gluteal muscle mass may be indicative reduced risk of insulin resistance which is commonly followed by development of T2D. The effect of gluteofemoral fat mass on metabolism has been investigated in physiological studies [51, 52]. A study by Manolopoulos et al [52] proposed that the fat mass in the gluteofemoral region traps the surfeit serum fatty acids which results in a low serum lipid levels. Furthermore, authors suggested that secreting adipokines leptin and adiponectin, the gluteofemoral fat mass might play a protective role in the development of T2D. Finally, Kuk et al. showed that in both sexes, for a given WC, higher HC is associated with higher gluteofemoral and abdominal subcutaneous fat mass and skeletal muscle while being associated with lower visceral fat mass [53].

Regarding the discriminatory power of anthropometric measures, in both sexes, among all the investigated anthropometric measures, WHtR had the highest prediction power, whereas HC had the lowest. Results of the current study are in line with several cohort studies [17, 22]. Previously, in short-term follow-up, we showed the superiority of WHtR to BMI in the prediction of T2D in both genders [54, 55]. The differences between our short versus long-term follow up studies might be due to different follow-up times, statistical approaches and covariates. Recently, a systematic review and meta-analysis by Ashwell et al [13] revealed a stronger association of WHtR with T2D rather than BMI in both genders.

In the current study, we looked for cut-points of different anthropometric measures using Youden’s index, which gives equal weight to both the sensitivity and specificity. Clinically, however, these may not be the appropriate cut-points since sensitivity versus specificity must be weighed against the seriousness of the disease, and the test under evaluation (whether it is a simple measurement or an invasive test and its cost). Hence, considering the coronary heart disease equivalency of T2D among Iranian population, [4] in line with the Inter99 Study, [16] we decided not to miss more than 25% of at-risk participants and fixed the sensitivity of all the anthropometric cut-points at about 75%; the cut-points resulted in specificities above 50% in all of the anthropometric measures except HC. Accordingly, for BMI, we recommend using 25.56 kg/m^2^ as a predictive cut-point in men and 27.12 kg/m^2^ in women. Data of the national non-communicable disease risk factors surveillance in Iran suggested cut-points close to the current study (24.8 kg/m^2^ in men and 26.3 kg/m^2^ in women) [56]. Assessing the accuracy of the ADA suggested cut-point [29] of BMI ≥ 25 kg/m^2^ in our population, its sensitivity and specificity were 89.2 and 37.5% in women and 78.8 and 46.7% in men, respectively which have noticeably lower specificities compared to our suggested cut-points. Our recommended cut-points for WC are 89 cm in men and 87 cm in women. Studies in different populations suggest different WC cut-points for predicting T2D which sounds reasonable due to ethnic differences [23, 24]. IDF suggested to use WC ≥ 94 cm and ≥ 80 cm in Middle-Eastern men and women, respectively [57]. Comparison between the false positive rates using our derived cut-points versus the ones suggested by IDF shows that our derived cut-point of 87 cm in women performed better (0.42 versus 0.66, respectively), however, in men, IDF cut-points showed lower false positive rates (0.47 and 0.30, respectively). But it should be considered that the sensitivity of WC cut-point suggested by IDF decreased to 54.2%; the issue which is not appropriate for screening T2D [16]. As a general finding in our study, when fixing the sensitivity at 75%, there was not a noticeable difference in specificity and PPV of the cut-points in men but in women, WHtR suggested cut-point had a higher specificity and PPV compared to other indices. Focusing on WHtR, we suggest the cut-point of 0.52 in men and 0.56 in women, results that somewhat support findings of a systematic review by Ashwell et al [13] to “keep your waist to less than half your height”. However, our derived cut-points showed a lower false positive rate both in men (0.46 versus 0.58) and women (0.38 versus 0.67).

Considering the limitation of the available data, [19, 20, 58] the joint analyses in the current study extend the understanding of the combined influence of obesity measures on incident T2D. In line with our study, Meisinger et al. showed that there are differences in the sex-specific relevance of measures of body fat distribution in predicting incident T2D [19]. In women, our findings suggested that different combinations of BMI-WC, BMI-WHtR, BMI-WHR, and BMI-HC (except high BMI-low WHtR and low BMI-high HC) were associated with high risk of developing T2D. In men, we showed that high BMI either in conjunction with high obesity indices or normal ones (except for normal HC) had a significant association with incidence of T2D in future, whereas those with normal BMI but high WHtR, WC or HC did not show a significant risk for the development of T2D. On the other hand, some studies proposed that BMI, WC, and WHR have very similar predictive powers for incident T2D [19, 20]. This difference may be explained by variation in methodological approaches and the sample size. It should be kept in mind that although from a statistical point of view some of the anthropometric measures have a stronger association with T2D and can predict it better, from a clinical perspective we cannot say that one obesity measure overweighs the other. It would be rational to ask physicians to refer those with higher than normal obesity indices no matter which index is high. Furthermore, it seems that combined high values of general and central obesity indices increase the specificity for the price of reduced sensitivity and PPV in predicting incident T2D.

Considering limitations, our results cannot be extrapolated to elder populations since participants of our study are adults, 20–60 years of age. In addition, excluding participants affected by conditions such as autoimmune diseases and acute or chronic infections which are known to impair glucose tolerance was not possible at baseline examination; however, those suffering from cancer, end-stage renal disease or cirrhosis were excluded. Likewise, we could not collect data regarding the use of estroprogestins in our participants, nevertheless, use of other drugs that have the potential to affect glucose impairment was carefully observed. Also, as shown in Table 1, respondent participants in our study generally had significantly higher levels of risk factors which might lead to overestimation of T2D incidence. These statistically significant differences might be attributed to large study sample size and they are not significant from the clinical point of view (e.g. mean of WHtR = 0.51 and 0.50 in respondents and non-respondents men, respectively). Moreover, almost all of the risk factors were adjusted in the multivariable models. Finally, unfortunately, HbA1C was not measured in TLGS participants; hence, we could not use HbA1c as a criterion for defining diabetic patients.

As for the strengths, our study included a large representative sample of Iranian adults in a sex-stratified population with reliable follow-up data. We had a reasonable number of events which allowed us to evaluate the long-term effects of anthropometric indices on T2D incidence. Also, we used both FPG and 2 h-PCPG as indicators of diabetes status both at baseline and follow-up examinations allowing us to have an accurate estimation of incident T2D.

## Conclusions

In conclusion, all anthropometric variables showed significant association with incident T2D considering a wide set of covariates including wrist circumferencein both sexes, except hip circumference in men. We showed that among all the obesity indices, in both sexes, WHtR performed the best while HC had the lowest prediction power. Fixing sensitivity at about 75%, not to miss more than 25% of at-risk individuals for development of T2D in long term, our derived cut-points for BMI, WC, WHtR, WHR, and HC in the Iranian population were 25.56 kg/m^2^, 89 cm, 0.52, 0.91, and 96 cm in men and 27.12 kg/m^2^, 87 cm, 0.56, 0.83, and 103 in women, respectively. Our derived cut-points for BMI and WHtR in both sexes, and WC in women are higher than the traditionally used cut-points, leading to a considerable reduction in false positive rates. Therefore, they might perform better if applied in the Iranian population. Further studies in other Iranian populations are required to check the external validity of our proposed cut-points in predicting development of T2D.

## Additional files

Additional file 1: The selection process of study sample to determine optimal cut-points for prediction of type 2 diabetes. Figure legend: The hatched (those who were free of T2D in the last follow-up examination, despite not participating in one or more follow-ups) and grey (those who developed T2D in each of the follow-up examinations) boxes indicate study sample.T2D, type 2 diabetes mellitus; Lost, lost to follow-up. (PDF 2766 kb)

Additional file 2: Baseline Characteristics of Respondents and Non-respondents, Tehran Lipid and Glucose Study (1999–2015). Table comparing baseline characteristics of respondents and non-respondents in the study. (DOCX 14 kb)

## Abbreviations

2 h-PCPG: 2 h-post-challenge plasma glucose
95% CIs: 95% confidence intervals
AUC: Area under the curve
BMI: Body mass index
DBP: Diastolic blood pressure
FPG: Fasting plasma glucose
HDL-C: High-density lipoprotein cholesterol
LMICs: Low- and middle-income countries
ROC: Receiver operating characteristics
SBP: Systolic blood pressure
SD: Standard deviation
T2D: Type 2 diabetes
TG: Triglycerides
TLGS: Tehran Lipid and Glucose Study
WC: Waist circumference
WHR: Waist to hip
WHtR: Waist to height ratios
