# Supplementary figures and images for: Optimal cut-points of different anthropometric indices and their joint effect in prediction of type 2 diabetes: results of a cohort study
Source: BMC Public Health. 2018 Jun 5;18:691. doi: 10.1186/s12889-018-5611-6 (PMC5987476; doi:10.1186/s12889-018-5611-6)

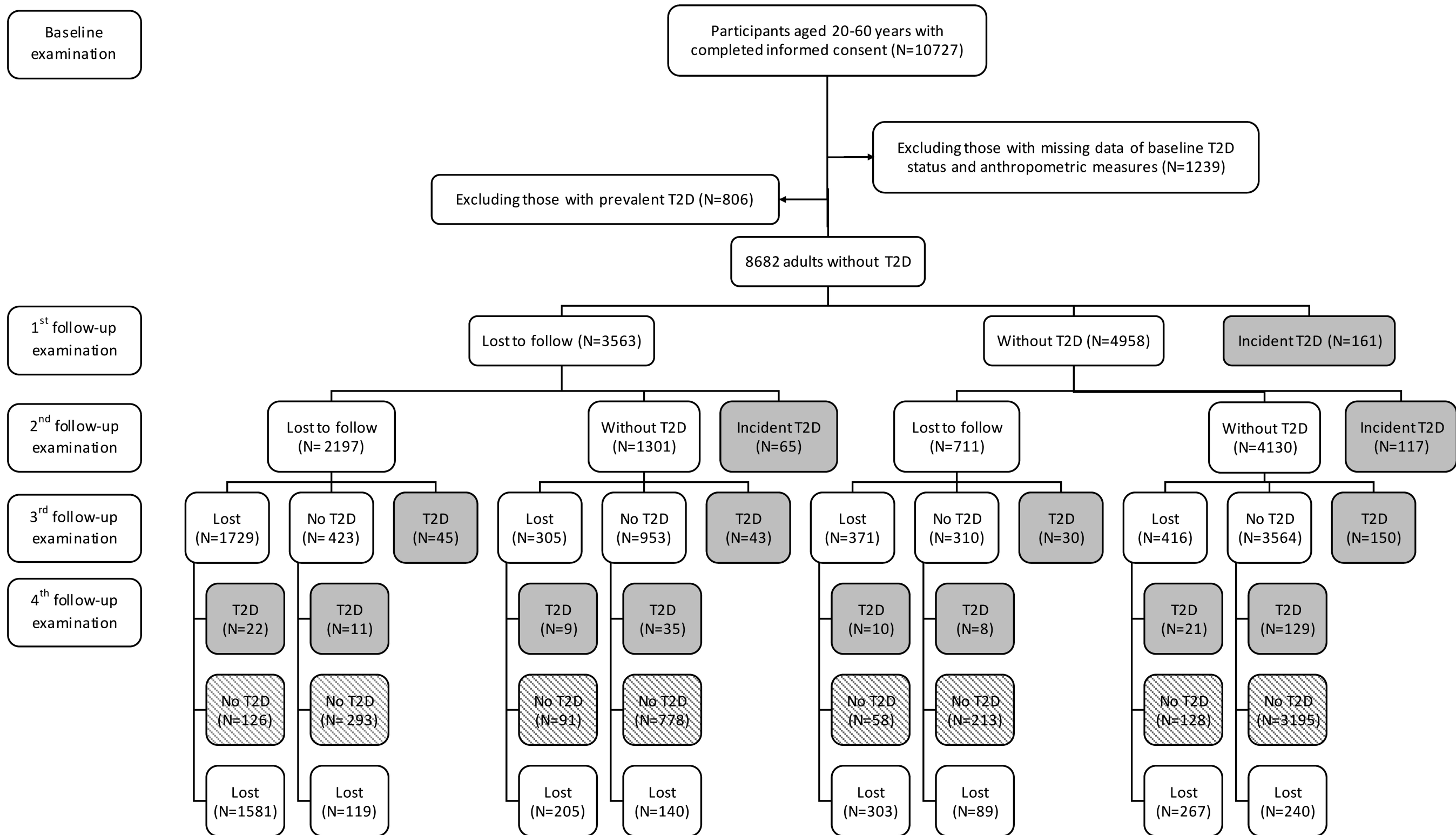

Supplement: Supplementary file 1 — The selection process of study sample to determine optimal cut-points for prediction of type 2 diabetes. Figure legend: The hatched (those who were free of T2D in the last follow-up examination, despite not participating in one or more follow-ups) and grey (those who developed T2D in each of the follow-up examinations) boxes indicate study sample.T2D, type 2 diabetes mellitus; Lost, lost to follow-up. (PDF 2766 kb) [file 12889_2018_5611_MOESM1_ESM.pdf]
