# Supplementary material for: Optimal cut-points of different anthropometric indices and their joint effect in prediction of type 2 diabetes: results of a cohort study
Source: BMC Public Health. 2018 Jun 5;18:691. doi: 10.1186/s12889-018-5611-6 (PMC5987476; doi:10.1186/s12889-018-5611-6)
Supplement: Supplementary file 2 — Baseline Characteristics of Respondents and Non-respondents, Tehran Lipid and Glucose Study (1999–2015). Table comparing baseline characteristics of respondents and non-respondents in the study. (DOCX 14 kb) [file 12889_2018_5611_MOESM2_ESM.docx]

**Baseline Characteristics^a^ of Respondents and Non-respondents,** **Tehran Lipid and Glucose Study (1999-2015)**

|  | **Respondent**  **(N= 7017)** | **Non-respondent**  **(N= 2904)** | **Difference (CI)** |
| --- | --- | --- | --- |
| Age (years) | 37.5 (10.3) | 33.8 (11.1) | 3.6 (3.2;4.1) |
| Family History of T2D; No. (%) | 1905 (27.1) | 682 (25.3) | 1.9 (0.0; 3.8) |
| Education Level; No. (%) |  |  |  |
| < 6 years | 1598 (22.8) | 572 (19.8) | 3.0% (1.2;4.7) |
| 6-12 years | 4290 (61.1) | 1885 (65.2) | -4.1% (-6.2; -2.0) |
| > 12 years | 1129 (16.1) | 433 (15.0) | 1.1% (-0.4;2.7) |
| Wrist Circumference (cm) | 16.6 (1.3) | 16.5 (1.3) | 0.1 (0.08;0.21) |
| WC (cm) | 87.0 (11.9) | 84.7 (12.7) | 2.3 (1.7;2.8) |
| Height (cm) | 163.2 (9.2) | 163.5 (9.3) | -0.3 (-0.7;0.1) |
| WHtR | 0.53 (0.08) | 0.52 (0.08) | 0.01 (0.011;0.018) |
| BMI (kg/m^2^) | 26.6 (4.6) | 25.9 (5.1) | 0.7 (0.4;0.9) |
| HC(cm) | 100.9 (9.3) | 99.8 (9.8) | 1.1 (0.6;1.5) |
| WHR | 0.86 (0.08) | 0.85 (0.09) | 0.01 (0.01;0.02) |
| SBP (mmHg) | 114.5 (15.0) | 112.9 (15.1) | 1.6 (0.9;2.2) |
| DBP (mmHg) | 76.1 (10.3) | 75.4 (10.3) | 0.7 (0.2;1.2) |
| FPG (mmol/L) | 4.95 (0.51) | 4.88 (0.54) | 0.06 (0.04;0.09) |
| TG/HDL-C | 0.38 (0.71) | 0.30 (0.70) | 0.08 (0.05;0.11) |

^a^ For continuous variables, values are presented as mean (SD) and difference [95% CI] was estimated using linear regression models. Categorical variables are presented as frequency (percentage) and difference [95% CI] was estimated by logistic regression.

CI, confidence interval; T2D, type 2 diabetes mellitus; WC, waist circumference; WHtR, waist to height ratio; BMI, body mass index; HC, hip circumference; WHR, waist to hip ratio; SBP, systolic blood pressure; DBP, diastolic blood pressure; FPG, fasting plasma glucose; TG/HDL-C, triglyceride to high density lipoprotein cholesterol ratio.
